# Complete and Assembled Genome Sequence of *Lactobacillus plantarum* RI-113 Isolated from Salami

**DOI:** 10.1128/genomeA.00183-17

**Published:** 2017-04-20

**Authors:** Raffael C. Inglin, Leo Meile, Jochen Klumpp, Marc J. A. Stevens

**Affiliations:** a Laboratory of Food Biotechnology, Institute of Food, Nutrition and Health, ETH Zurich, Zurich, Switzerland; b Laboratory of Food Microbiology, Institute of Food, Nutrition and Health, ETH Zurich, Zurich, Switzerland

## Abstract

We present here the complete genome sequence of *Lactobacillus plantarum* RI-113, a strain isolated from salami, which was determined using single-molecule real-time sequencing.

## GENOME ANNOUNCEMENT

*Lactobacillus plantarum* strains have been isolated from a broad spectrum of ecosystems such as silage, olives, sourdough, sauerkraut, cheese, and fermented sausages (1, 2). This habitat diversity of *L. plantarum* might be related to abundant gene functions resulting in a genome size which is one of the largest among lactobacilli (3, 4). *L. plantarum* RI-113 is a single-colony strain isolated from salami that grows at a pH of 3.5, 7.5% NaCl, and 5% ethanol at a temperature range of 14°C to 43°C in Man-Rogosa-Sharpe medium. The strain shows antifungal activity against *Trichosporon* sp. and *Rhodotorula mucilaginosa*, as detected in a high-throughput screening (5). Genomic DNA was isolated by first using lysozyme-based cell-wall digestion with a Wizard genomic DNA purification kit (Promega, Dübendorf, Switzerland). The genome was sequenced using single–molecule real-time sequencing cells on a PacBio RS II platform (Pacific Biosciences, Menlo Park, CA, USA) at the Functional Genomics Center Zurich (Zurich, Switzerland). In total, 94,382 reads, with a mean length of 12,974 bp resulting in 370× coverage, were assembled into a single contig and six plasmids using the Hierarchical Genome Assembly Process (6). The genome was automatically annotated using the NCBI Prokaryotic Genome Annotation Pipeline. The genome of *L. plantarum* RI-113 consists of a 3,462,990-bp circular molecule and comprises 67 tRNA genes and 16 rRNA genes. The G+C content of the genome is 44.34%, and a total of 3,361 protein-coding sequences were predicted.

### Accession number(s).

Sequence and annotation data of the complete *L. plantarum* strain RI-113 genome have been deposited at GenBank under the accession numbers CP017406 (genome) and CP017407 to CP017412 (six plasmids).
